# Awareness of Golden Proportion in Tooth Forms Among Dental Scholars in Saudi Arabia: A Cross-Sectional Study

**DOI:** 10.7759/cureus.40619

**Published:** 2023-06-19

**Authors:** May W Al-Khudhairy, Mahesh Suganna, Dalal Talal Aldhafeeri, Ahoud Mohammed Alshehri, Hessah Adel Alharbi, Rahaf Sulaiman Alenezi, Areej Mubarak Bakheit

**Affiliations:** 1 Department of Oral Biology, College of Applied Medical Sciences, Riyadh Elm University, Riyadh, SAU; 2 Department of Prosthodontics, Riyadh Elm University, Riyadh, SAU; 3 Department of Dentistry, College of Dentistry, Riyadh Elm University, Riyadh, SAU

**Keywords:** dentistry, dental scholars, tooth forms, cross-sectional study, gop

## Abstract

Background

There are numerous uses of golden proportion (GoP) in dentistry, particularly cosmetic dentistry. The research on GoP has been lacking, particularly the knowledge of GoP among dentistry students still enrolled in classes and those undergoing training. Therefore, this study primarily aimed to determine how knowledgeable dental scholars (dentist scholars are final-year undergraduates, interns, Ph.D. scholars, and postgraduate students) in Saudi Arabia were of GoP in tooth forms.

Methodology

A total of 500 scholars who met the requisite criteria were considered eligible for this study and were provided with a questionnaire that consisted of background questions and 16 close-ended questions related to GoP. The data was analyzed, and descriptive and inferential statistics were used, with a *P*-value ≤ 0.05 at a 95% confidence interval (CI) deemed statistically significant.

Results

It was observed that most respondents believed that a smile was important for a patient. The results also indicated that most respondents had heard of GoP for smile designing, indicating that it is a well-known concept among dental scholars in Saudi Arabia. The findings also revealed that most respondents believed that the GoP ratio is 1.618 and that it is important as a guide to anterior restoration.

Conclusions

Most respondents considered the smile important and the golden ratio to be present in many fields, but their understanding of the concept varied. However, the study had some limitations, including the potential bias in self-reported responses and a lack of clinical application of the GoP. Future studies could investigate the practical implications of GoP in aesthetic dentistry and the effect of demographic factors on awareness and understanding of the concept.

## Introduction

Golden proportion (GoP), or the golden ratio, is a mathematical ratio of approximately 1.618 [1]. It is a phenomenon observed in various natural and human-made objects and has been used in art and design for centuries [1]. In dentistry, GoP describes the ideal proportion of teeth to create a natural and aesthetically pleasing smile [2]. This is achieved by using the ratio to determine the ideal proportions between the width and length of teeth, as well as the proportion between adjacent teeth [3]. GoP exists in the dimensions of the human face, particularly in the relationship between the width of the mouth and the distance between the eyes, and has served as a guide for facial reconstruction and orthodontic treatment planning [4].

GoP has numerous applications in dentistry, particularly aesthetic dentistry [5]. One of the most significant applications is designing smile makeovers, where GoP serves as a guide for tooth size and proportion [4]. It is believed that GoP is an essential factor in achieving aesthetically pleasing and natural-looking smiles [5]. Dentists also use GoP to guide anterior restorations, ensuring that the size and shape of the teeth are in proportion with each other and the facial features of the patient [3]. Additionally, GoP is used in the placement of dental implants, as it helps ensure proper alignment and balance with adjacent teeth [6]. GoP is also used to determine the ideal width-to-height ratio for teeth, which can be useful in diagnosing and treating certain dental conditions such as bruxism and malocclusion [7]. Furthermore, GoP is used to fabricate dental prostheses such as dentures to ensure that the artificial teeth are in proportion with the natural teeth and the facial features of the patient [8-9]. Overall, the applications of GoP in dentistry are diverse and significant, and its use can greatly enhance the aesthetic and functional outcomes of dental treatments.

However, the literature on this topic has been lacking, especially about the knowledge of GoP among dentists and undergraduates still undergoing study and training. Therefore, the primary objective of this study was to investigate the awareness of GoP in tooth forms among dental scholars in Saudi Arabia. The intended outcomes of this study included baseline data and the identification of data gaps that might help with planning, implementing, and evaluating the practice toward developing a geometric or mathematical proportion to relate the successive width of anterior teeth among dentists and undergraduates in Saudi Arabia.

## Materials and methods

Study design

This study underwent a thorough review by the Institutional Review Board (IRB) of Riyadh Elm University. The research questions were formulated to assess the level of awareness of GoP in tooth forms among dental scholars in Saudi Arabia. The first research question was about determining whether dental students in Saudi Arabia were aware of GoP in tooth forms, while the second research question investigated whether awareness varied among dentists and undergraduates in Saudi Arabia.

Hypothesis for the study

The null hypothesis stated that the awareness of GoP in tooth forms was comparable among dental scholars in Saudi Arabia. However, the alternative hypothesis suggested that awareness varied among dentists and undergraduates in Saudi Arabia.

Study domain

The study was conducted and considered respondents from 10 different regions of Saudi Arabia. The primary aim for selecting the varied provinces was to avoid selection bias and ensure the representation of sampling units from the entire country of Saudi Arabia. This criterion was applied for the generalizability of study findings and not for comparison between the provinces, which is beyond the purpose of the study.

Study subjects and groups

The probability sampling technique was applied to enroll the study subjects randomly. The study group included a comparison of the awareness of GoP in tooth forms among different study groups, with the groups comprising final-year undergraduates, interns, postgraduates, and Ph.D. scholars from both government and private dental schools in Saudi Arabia.

Data collection

The questionnaire for this study was developed from a previously published study [10] and was modified and pretested by the Department of Prosthodontics faculty. The validity of the questionnaire was determined by the pilot study. A pilot trial of 20 participants from the target population was done to assess the clarity and reliability of the questionnaire with a content validity index of 1 and Cronbach's alpha value of 0.8.

The questionnaire consisted of (1) background questions regarding age, gender, and educational status and (2) 16 close-ended questions related to the GoP, the GoP ratio, smile designing, the GoP ratio in the determination of the facial aspects, and the instrument used for measurement where the resposes were scored based on the descriptive analysis of the responses recieved.

A list of all the dental colleges in Saudi Arabia was obtained from the Ministry of Education portal, and a few governments and private dental schools were selected using a simple random sampling technique. The Dean for Academic Affairs was contacted to send the link to all the targeted participant groups via email to facilitate their early participation only once per participant.

Based on previously reported studies [11], a sample size of 500 was considered adequate and participants were recruited until that number was reached. The voluntary participation and confidentiality of study subjects were duly ensured, and the respondents remained completely anonymous.

Inclusion and exclusion criteria

Inclusion criteria were undergraduates, postgraduates, and residents from various dental colleges in Saudi Arabia. Students who were willing to participate voluntarily were included in this study, and their confidentiality was ensured. The online questionnaire was distributed electronically using a link generated using the Google Docs service. First-, second-, and third-year dental undergraduate studies were not selected for this study. Those individuals who declined to participate or answer the questionnaire were considered ineligible for this study.

Data management and statistical protocol

Data was entered and analyzed using IBM Statistical Package for Social Sciences (SPSS) Statistics for Windows, version 25.0 (IBM Corp., Armonk, NY, USA). A descriptive analysis of the data was followed by inferential statistics. Chi-square and Fisher's exact tests were used to compare categorical data. A *P*-value ≤ 0.05 at the 95% confidence interval (CI) was deemed statistically significant.

## Results

Of the 500 people provided with the questionnaire, 350 responded, indicating a 70% response rate. Table 1 provides information on gender distribution across different categories of individuals.

**Table 1:** Age distribution of the respondents as per their gender and educational status.

Age category and educational status	Females	Males	Grand total
23-25 years	86	26	112
Final-year undergraduates	46	10	56
Interns	31	9	40
Ph.D. scholars	4	1	5
Postgraduates	5	6	11
26-28 years	80	68	148
Final-year undergraduates	19	21	40
Interns	25	22	47
Ph.D. scholars	12	6	18
Postgraduates	24	19	43
29-31 years	17	26	43
Final-year undergraduates	3	1	4
Interns	1	2	3
Ph.D. scholars	3	4	7
Postgraduates	10	19	29
32-34 years	3	14	17
Interns		1	1
Ph.D. scholars		5	5
Postgraduates	3	8	11
35-37 years	5	4	9
Final-year undergraduates	1		1
Ph.D. scholars	1	2	3
Postgraduates	3	2	5
38-40 years	4	3	7
Ph.D. scholars	3		3
Postgraduates	1	3	4
41-43 years	4		4
Final-year undergraduates	3		3
Ph.D.	1		1
44-46 years	3	4	7
Final-year undergraduates	2	2	4
Ph.D. scholars	1	1	2
Postgraduates		1	1
47-49 years		1	1
Postgraduates		1	1
53-55 years		1	1
Final-year undergraduates		1	1
56-58 years	1		1
Final-year undergraduates	1		1
Grand total	203	147	350

The categories are represented in the rows, and the genders (male and female) are represented in the columns. Table 2 provides the number of individuals in each category and gender, while we provide the statistical discussion in terms of the percentages.

**Table 2:** Number of respondents as per their respective age.

Age (years)	Frequency	Percentage (%)	Valid percentage (%)	Cumulative percentage (%)
23	25	7.1	7.1	7.1
24	44	12.6	12.6	19.7
25	43	12.3	12.3	32
26	52	14.9	14.9	46.9
27	85	24.3	24.3	71.1
28	11	3.1	3.1	74.3
29	19	5.4	5.4	79.7
30	20	5.7	5.7	85.4
31	4	1.1	1.1	86.6
32	5	1.4	1.4	88
33	10	2.9	2.9	90.9
34	2	0.6	0.6	91.4
35	4	1.1	1.1	92.6
36	2	0.6	0.6	93.1
37	3	0.9	0.9	94
38	1	0.3	0.3	94.3
39	2	0.6	0.6	94.9
40	4	1.1	1.1	96
42	2	0.6	0.6	96.6
43	2	0.6	0.6	97.1
45	4	1.1	1.1	98.3
46	3	0.9	0.9	99.1
48	1	0.3	0.3	99.4
55	1	0.3	0.3	99.7
57	1	0.3	0.3	100
Total	350	100	100	

Table 3 is a representation of the various questions that were asked and the responses that were obtained.

**Table 3:** Frequency and percentages pertaining to the questionnaire items as answered by the respondents. GoP, golden proportion

Questionnaire item	Variable assessed	Frequency	Percentage (%)	Valid percentage (%)	Cumulative percentage (%)
Q1. How important is a smile for a patient?	Moderately important	31	8.9	8.9	98.6
	Less important	5	1.4	1.4	100
	Total	350	100	100	
Q2. The golden ratio is claimed to appear in many fields, such as cosmology, theology, arts, architecture, botany, and others.	True	242	69.1	69.1	69.1
	False	30	8.6	8.6	77.7
	Do not know	78	22.3	22.3	100
	Total	350	100	100	
Q3. Have you ever heard of GoP for smile designing?	Yes	261	74.6	74.6	74.6
	No	89	25.4	25.4	100
	Total	350	100	100	
Q4. What is the GoP in tooth forms?	Ratio between two teeth	283	80.9	80.9	80.9
	Fraction between two teeth	67	19.1	19.1	100
	Total	350	100	100	
Q5. Do you believe that GoP exists between the larger and smaller lengths of a tooth?	Yes	235	67.1	67.1	67.1
	No	34	9.7	9.7	76.9
	Do not know	81	23.1	23.1	100
	Total	350	100	100	
Q6. What do you think is the GoP ratio?	1:212	109	31.1	31.1	31.1
	1:618	201	57.4	57.4	88.6
	1:445	40	11.4	11.4	100
	Total	350	100.0	100.0	
Q7. What is determined with the help of GoP?	Aesthetics	98	28	28	28
	Facial form	32	9.1	9.1	37.1
	Size of tooth	42	12	12	49.1
	All of the above	178	50.9	50.9	100
Q8. Does GoP help in smile design?	Yes	284	81.1	81.1	81.1
	No	27	7.7	7.7	88.9
	Do not know	39	11.1	11.1	100
	Total	350	100	100	
Q9. Do you believe that GoP is important as a guide to anterior restoration?	Yes	269	76.9	76.9	76.9
	No	34	9.7	9.7	86.6
	Do not know	47	13.4	13.4	100
	Total	350	100	100	
Q10. Does tooth rotation, crowding, or spacing affect GoP?	Yes	253	72.3	72.3	72.3
	No	36	10.3	10.3	82.6
	Do not know	61	17.4	17.4	100.0
	Total	350	100.0	100.0	
Q11. Do all age groups have a constant GoP?	Yes	124	35.4	35.4	35.4
	No	154	44.0	44.0	79.4
	Do not know	72	20.6	20.6	100.0
	Total	350	100.0	100.0	
Q12. Do you believe that size and shape of the tooth affect GoP?	Yes	279	79.7	79.7	79.7
	No	26	7.4	7.4	87.1
	Do not know	45	12.9	12.9	100.0
	Total	350	100.0	100.0	
Q13. Which tooth has the highest GoP ratio?	Maxillary Centrals	237	67.7	67.7	67.7
	Maxillary Laterals	44	12.6	12.6	80.3
	Maxillary Canine	69	19.7	19.7	100.0
	Total	350	100.0	100.0	
Q14. Which of the below device can be used to calculate the GoP?	Vernier caliper	194	55.4	55.4	55.4
	Scale	68	19.4	19.4	74.9
	Compass	28	8.0	8.0	82.9
	None of the above	60	17.1	17.1	100.0
	Total	350	100.0	100.0	
Q15. Do you agree GoP must be measured from the mesial contact point?	Yes	226	64.6	64.6	64.6
	No	42	12.0	12.0	76.6
	Do not know	82	23.4	23.4	100.0
	Total	350	100.0	100.0	
Q16. Do you agree that knowledge and information on GoP could be useful in improving the field of aesthetic dentistry?	Yes	272	77.7	77.7	77.7
	No	35	10.0	10.0	87.7
	Do not know	43	12.3	12.3	100.0
	Total	350	100.0	100.0	

Out of the 350 respondents, the majority (98.6%) felt that a smile is moderately important for a patient, while a small percentage (1.4%) thought that it is less important. Nearly 69.1% of the respondents believed that the golden ratio appears in many fields, whereas 8.6% disagreed. Around 22.3% of the respondents did not know about the golden ratio. About 74.6% of the respondents had heard of GoP for smile designing, while 25.4% had not. Most respondents (80.9%) thought that GoP in tooth forms is the ratio between two teeth, while the remaining respondents (19.1%) believed that it is a fraction between two teeth. Most respondents (67.1%) believed that GoP exists between the larger and smaller lengths of a tooth. A small percentage of respondents (9.7%) did not believe in the existence of GoP, while 23.1% did not know.

The responses to the following question varied among the respondents: *What do you think is the GoP ratio?* However, most respondents (57.4%) believed that the GoP ratio was 1.618; followed by 31.1%, who thought it was 1.212; and 11.4%, who believed it was 1.445. When asked about the role of GoP, most respondents (50.9%) believed that it helps determine the following: aesthetics, facial form, and tooth size. Additionally, 28.0% of respondents believed that it helps in the determination of aesthetics, 12.0% thought it helps in the determination of the size of the tooth, and 9.1% believed it helps in the determination of facial form. Most respondents (81.1%) believed that GoP helps in smile design, while only 7.7% disagreed. Around 11.1% of the respondents did not know about the role of GoP in smile design. Of the respondents, 76.9% believed that GoP is important as a guide to anterior restoration, while 9.7% did not believe and 13.4% did not know. Of the respondents, 72.3% believed that tooth rotation, crowding, or spacing affect the GoP ratio, while 10.3% did not and 17.4% did not know. A total of 35.4% of the respondents believed that all age groups have a constant GoP, while 44.0% did not believe and 20.6% did not know. A total of 79.7% of the respondents believed that the size and shape of the tooth affect the GoP, while 7.4% did not believe and 12.9% did not know. Of the respondents, 67.7% believed that the maxillary centrals have the highest GP ratio, while 12.6% believed it is the maxillary laterals, and 19.7% believed it is the maxillary canine. Of the respondents, 55.4% believed that the vernier caliper can be used to calculate the GoP ratio, while 19.4% believed it is a scale, 8.0% believed it is a compass, and 17.1% believed none of the above can be used.

Of the respondents, 64.6% agreed that GoP must be measured from the mesial contact point, 12% did not believe, and 23.4% did not know. Of the respondents, 77.7% agreed that knowledge and information on GoP could be useful in improving the field of aesthetic dentistry, while 10% did not believe and 12.3% did not know.

## Discussion

The findings of this study provide important insights into the awareness of GoP in tooth forms among dental scholars in Saudi Arabia. The study found that most respondents believed that a smile was moderately important for a patient, highlighting the importance of aesthetics in dental practice. Furthermore, the results indicated that most respondents had heard of GoP for smile designing, indicating that it is a well-known concept among dentists and undergraduates in Saudi Arabia. The findings also revealed that most respondents believed that the GoP ratio is 1.618 and that it is important as a guide to anterior restoration. This knowledge is crucial for dental practitioners to provide quality care to their patients. Moreover, most respondents believed that GoP helps in determining aesthetics, facial form, and the size of the tooth, highlighting the significance of GoP in dental aesthetics. It was also revealed that knowledge and information on GoP could be useful in improving the field of aesthetic dentistry. This indicates that educational programs focusing on GoP could help dentists and undergraduates understand aesthetic dentistry better and improve the quality of dental care provided to patients. The specific gaps in the literature that this study filled include the lack of knowledge regarding the awareness of GoP among dental dentists and undergraduates in Saudi Arabia. Previous studies have explored the concept of GoP in dentistry, but this study specifically focused on the awareness of dentists and undergraduates in Saudi Arabia. The study also investigated the extent to which dentists and undergraduates are aware of the application of GoP in tooth forms and smile design. The findings of the study provide useful information on the awareness of the GoP in tooth forms among dentists and undergraduates in Saudi Arabia. Dental educators can use this information to develop educational programs for undergraduates that emphasize an understanding of GoP and its role in dental aesthetics. Moreover, this study can be used as a foundation for further research on the topic to better understand the role of GoP in dental aesthetics and its impact on patient outcomes.

Numerous studies have argued that GoP is more of a theoretical idea and may not even be necessary for the perception of an attractive smile [12-15]. A study concluded that GoP was not even a common element in attractive smiles from their investigation [16]. Since the GoP tends to produce smaller-than-usual canines and maxillary incisors, early research claimed that it could only be applied to the anterior teeth within specific parameters [17-19]. According to a previously published study on people in the southwest of Saudi Arabia, GoP existed between the canines of males and the incisors of females [6].

However, GoP or any other specified percentage should not be utilized blindly during anterior prosthodontic restorations because it may, in some cases, provide unattractive outcomes. Instead of adhering to a universal norm, anterior dental aesthetics should be restored with distinctive and suitable proportions based on individual cultural traits and actual local measurements [20]. The same finding was observed in another study [21]. This view is supported by the findings of various studies [12-14]. For instance, the proportion of related aesthetics in a North American population for incisors was reported to be 66% and that of canines was 84% [17]. The proportion of related aesthetics was reported to be 66% for incisors and 70% for canines in a population of Nepalese people. The authors even suggested this proportion as a standard for dental care in the maxillary anterior region in populations of the same region [21]. Moreover, when planning for prosthodontic treatment, particularly in the aesthetic zone, restoring symmetry is also necessary and may even be more crucial than establishing GoP [22].

There are several limitations to this study. First, the study only focused on dentists and undergraduates in Saudi Arabia, which may not be representative of the larger population of dentists or undergraduates in other countries. Also, the study relied on self-reported responses from participants, which may be subject to bias and inaccuracies. Moreover, the study did not assess the knowledge and understanding of the participants beyond the questions asked, which may limit their overall understanding of GoP. Additionally, the study did not investigate the impact of demographic factors such as age, gender, and educational background on the awareness and understanding of the GoP among participants. Moreover, the fact that questionnaires were sent till 500 responses were obtained could be said to be another limitation. Finally, the study did not assess the practical application of the GoP in clinical settings, which may be an important factor in determining its value in aesthetic dentistry.

## Conclusions

This study aimed to investigate the awareness and understanding of GoP in tooth forms among dentists and undergraduates in Saudi Arabia. The study found that most respondents considered a smile to be important for a patient and the golden ratio to be present in many fields. Most respondents had heard of the GoP for smile designing, but there was some variation in their understanding of the concept. Respondents generally agreed that GoP was important in smile design and anterior restoration. However, the study also revealed several limitations, including potential bias in self-reported responses and a lack of practical application of GoP in clinical settings. Future studies could further investigate the practical implications of GoP in aesthetic dentistry and explore the impact of demographic factors on awareness and understanding of the concept. Overall, this study provides valuable insights into the current state of awareness and understanding of the GoP among dentists and undergraduates in Saudi Arabia.
